# A Parcellation Based Nonparametric Algorithm for Independent Component Analysis with Application to fMRI Data

**DOI:** 10.3389/fnins.2016.00015

**Published:** 2016-01-29

**Authors:** Shanshan Li, Shaojie Chen, Chen Yue, Brian Caffo

**Affiliations:** ^1^Department of Biostatistics, Indiana University Fairbanks School of Public Health, Indiana UniversityIndianapolis, IN, USA; ^2^Department of Biostatistics, Johns Hopkins Bloomberg School of Public Health, Johns Hopkins UniversityBaltimore, MD, USA

**Keywords:** blind source separation, density estimation, functional MRI, p-spline bases, signal processing

## Abstract

Independent Component analysis (ICA) is a widely used technique for separating signals that have been mixed together. In this manuscript, we propose a novel ICA algorithm using density estimation and maximum likelihood, where the densities of the signals are estimated via p-spline based histogram smoothing and the mixing matrix is simultaneously estimated using an optimization algorithm. The algorithm is exceedingly simple, easy to implement and blind to the underlying distributions of the source signals. To relax the identically distributed assumption in the density function, a modified algorithm is proposed to allow for different density functions on different regions. The performance of the proposed algorithm is evaluated in different simulation settings. For illustration, the algorithm is applied to a research investigation with a large collection of resting state fMRI datasets. The results show that the algorithm successfully recovers the established brain networks.

## 1. Introduction

This manuscript puts forward two innovations. Firstly, we demonstrate a fast, likelihood motivated and straightforward method for applying independent components analysis (ICA). Secondly, we propose a parcellation based adjustment when the source signals distribute differently across regions. Our work is routed in the context of understanding human brain networks, and we use functional magnetic resonance imaging (fMRI) data for illustration in this manuscript.

We approach our study of fMRI by simultaneously analyzing all voxels. This is in contrast to regional or seed-based approaches (Buckner et al., 2005; Wang et al., 2006; Allen et al., 2007) that restrict attention to carefully chosen locations. Such approaches require strong assumptions on the choice of seeds or parcellation used to define region. Hence voxel-wise approaches are important complementary procedures. Given the volume of voxels under study (usually on the order of fifty thousand non-background ones), flexible yet parsimonious models approaches are required. However, even with parsimonious models, whole brain voxel-level techniques are more empirical and exploratory than their more hypothesis driven regional and seed-based counterparts. Thus, exploratory factor-analytic models are common approaches in voxel-level investigations.

Independent components analysis (ICA) is a factor-analytic approach that has been frequently utilized for the analysis of functional neuroimaging data, because of its success in discovering important brain networks in many applications (McKeown et al., 1998a,b; Calhoun et al., 2001a; Guo and Pagnoni, 2008). Two key benefits of ICA are its exploratory nature and its often considered reasonable underlying generative model. Specifically, it models collected signals, *X*, as linear weighted combinations of independent sources, *S*_1_, *S*_2_, …*S*_*p*_. Thus, we can write the noise-free ICA model as *X* = *AS*, where *S* = [*S*_1_, *S*_2_, …, *S*_*Q*_] and *A* is a *Q* × *Q* full rank matrix, the so-called mixing matrix. The goal of ICA is to recover the underlying signals *S*_1_, *S*_2_, …, *S*_*Q*_ from their observed mixtures *X*_1_, *X*_2_, …, *X*_*Q*_. Note that, in the context of fMRI, the independent components *S*_1_, *S*_2_, …, *S*_*Q*_ are often interpreted as brain networks and *A* is the mixing matrix characterizing the temporal pattern of the corresponding brain networks.

Various algorithms for ICA have been proposed in the literature. See Hyvärinen et al. (2001); Comon and Jutten (2010); Risk et al. (2014) for comprehensive reviews. One common procedure is to postulate a parametric family for the source distributions and then obtain the independent components (ICs) by optimizing a contrast function that measures the distribution property of the output (Samworth and Yuan, 2012). The contrast functions can be selected based on different measures, such as entropy, mutual independence, high-order decorrelations, divergence between the joint distribution of the output and some model, etc. (Cardoso, 1998). These include the popular FastICA algorithm (Hyvärinen and Oja, 2000), the JADE algorithm (Cardoso, 1999), the Pearson ICA algorithm (Karvanen et al., 2000), and a few other algorithms proposed by Comon (1994); Amari and Cardoso (1997); Li and Adali (2010). An alternative procedure is to assume smooth densities for the source distributions and use nonparametric or semiparametric approaches to estimate those density functions. The mixing matrix can then be derived using maximum likelihood method. For example, Bach and Jordan (2003) developed a nonparametric estimation approach based on canonical correlations in a reproducing kernel Hilbert space. Hastie and Tibshirani (2002) expressed the source distribution by an exponentially tilted Gaussian function and used the fixed-point algorithm for estimation of the mixing matrix. Boscolo et al. (2004) used kernel density estimation techniques to model the underlying densities and quasi-Newton method for optimization. Guo and Pagnoni (2008) used Gaussian mixture models for the source distribution and provided an expectation-maximization (EM) framework for estimation, assuming Gaussian noise in the model. Eloyan et al. (2013) estimated the source distribution by using mixture density estimates, and proposed a constrained EM algorithm for estimation.

The benefit of the likelihood-based ICA algorithm is that, as a byproduct of the ICA algorithm, one obtains the fully specified likelihood of the ICA model which can be used for further statistical inference. For example, based on the fully specified likelihood, one can conduct Bayesian analysis or perform likelihood based model selection. However, the existing likelihood-based ICA algorithms are mostly semi-parametric and are usually computationally intensive. In this manuscript, we aim to develop a likelihood-based algorithm that is exceedingly simple and truly blinded to the source distributions.

We propose to estimate the density function of the ICs via histogram smoothing, following a well-known approach in the penalized spline literature. At its core, likelihood-based ICA requires estimation of the mixing matrix and flexible density estimation for the ICs. Our approach, like many other likelihood-based approaches, iteratively estimates these components separately using block maximization. In contrast to other approaches, we use an exceedingly simple density estimation technique via histogram smoothing. Specifically, we assume the bin counts of the frequency histogram follow a Poisson distribution and express the mean counts as sum of B-spline bases via generalized linear model. To smooth the histogram, we follow Eilers and Marx (1996) to construct a penalized likelihood with a difference penalty on coefficients of adjacent B-splines. Apart from its simplicity, a benefit of this approach is speed. Density estimation and evaluation for tens of thousands of voxels is time consuming, and worse, is performed within an iterative algorithm. Using histogram smoothing, the voxel-level calculation reduces to estimating a histogram, a very fast process.

We briefly mention that, in our primary area of application, fMRI, we focus entirely on noise-free group spatial independent component analysis. By assuming noise-free model, noise in the data is absorbed into the estimated ICs and the mixing matrix. By using spatial ICA model, the fMRI data is decomposed into spatial maps multiplied by their respective time courses, where the maps are drawn from spatial distributions that are statistically independent (Calhoun et al., 2001a). The spatial independence assumption is well suited to the sparse nature of the spatial pattern for typical brain activation (McKeown and Sejnowski, 1998; Guo and Pagnoni, 2008). The time courses estimated from spatial ICA describe the temporal characteristics of functional networks, i.e., areas of temporal correlation in the BOLD signal. For multi-subject fMRI data, we assume common spatial maps for all subjects and subject-specific mixing matrices, therefore, we can concatenate all subjects' data in the temporal domain, and apply ICA to the aggregated data matrix. The group mixing matrix is the concatenated time course for all subjects. Individual mixing matrices can be backreconstructed by partitioning the group mixing matrix into submatrices corresponding to each subject.

The remainder of the paper is organized as follows. Section 2 describes the p-spline based ICA algorithm and considers relaxation of the *i.i.d* signal assumption. Section 3 shows the performance of the proposed algorithm in simulation study. Section 4 provides the application of the proposed algorithm to the 1000 Functional Connectome Project (https://www.nitrc.org/projects/fcon_1000/), while Section 5 gives a discussion.

## 2. Methods

### 2.1. Description of ICA methodology

Independent component analysis models collected signals as linear weighted combinations of independent sources. Notationally, let *X*_*i*_ be a *T* × *V* matrix for subject *i* = 1, …, *I*. In the context of fMRI, *T* indicates scans while *V* indicates voxels. Assume the number of ICs is *Q*. The ICA model specifies *X*_*i*_ = *A*_*i*_*S*, where *A*_*i*_ is a *T* × *Q* mixing matrix and *S* is a *Q* × *V* matrix of ICs. By assuming common spatial maps across subjects, we can stack the individual matrices in the temporal domain. Let $X={\left[X_{1}^{T},X_{2}^{T},\ldots ,X_{I}^{T}\right]}^{T}$ be the *TI* × *V* group data matrix, and $A={\left[A_{1}^{T},A_{2}^{T},\ldots ,A_{I}^{T}\right]}^{T}$ be the *TI* × *Q* group mixing matrix. Spatial group ICA simply specifies the standard model

(1) $$\begin{array}{l} X=AS. \end{array}$$

We use parentheses to index matrices so that *X*(*t, v*) is element (*t, v*) of *X* and define *X*(*t*, ·) as row *t* of *X* and *X*(·, *v*) as column *v*. Then, model (1) could be rewritten as $X\left(t,v\right)=\overset{Q}{\underset{q=1}{\sum }}A\left(t,q\right)S\left(q,v\right)$ and $X=\overset{Q}{\underset{q=1}{\sum }}A\left(\cdot ,q\right)S\left(q,\cdot \right).$

We assume that *E*[*X*] = μ_*x*_ = 0 and hence *E*[*S*] = μ_*s*_ = 0. If this assumption were not made, the ICA model would imply *X* − μ_*x*_ = *A*(*S* − μ_*S*_), which is exactly an ICA model with a centered data matrix and the ICs having mean 0. Hence, *X* is demeaned prior to analyses and μ_*S*_ is assumed to be zero. Similarly, since *A*(·, *q*)*S*(*q*, ·) = {*A*(·, *q*)∕*c*} ^*^ {*cS*(*q*, ·)}, ICs are only identified up to scalar multiplication. Thus, we assume that Var{*S*(*q, v*)} = 1 for *q* = 1, …, *Q* and *v* = 1, …, *V*.

ICA gets its name by assuming that *S*(*q*, ·) ⫫ *S*(*q*′, ·) when *q* ≠ *q*′, where ⫫ implies statistical independence. However, standard variations of ICA also assumes that ${\left\{S\left(q,v\right)\right\}}_{v=1}^{V}$ is an *i.i.d* collection, which we also adopt for now. The *i.i.d* assumption will be relaxed later in the next subsection. As a consequence of these assumptions, *X*(·, *v*) ⫫ *X*(·, *v*′) when *v* ≠ *v*′; yet note that *X*(*t*, ·) is not (necessarily) independent of *X*(*t*′, ·).

Typically, *Q* < *TI* and Equation (1) is overdetermined. A two-stage dimension reduction is often performed to reduce the computational load and avoid overfitting (Calhoun et al., 2001a; Beckmann and Smith, 2005; Guo and Pagnoni, 2008; Eloyan et al., 2013; Risk et al., 2014). Specifically, in the first stage, an SVD is performed in the temporal domain within subject, where the first *R* eigenvectors are retained. The dimension for the group data matrix then becomes *RI* × *V*. In the second stage, an SVD is performed on the group data matrix obtained from the first stage and the first *Q* eigenvectors are retained to force a determined linear system for the group ICA model. This discards information in the data. However, one hopes that by selecting the first *Q* singular vectors, the most relevant features of the data will be retained. The choice of *R* and *Q* could be based on various criteria, including variance explained, information-theoretic criteria, and practical considerations. This is not a major concern in this article.

### 2.2. ICA through fast nonparametric density estimation

ICA estimates *S* by seeking an unmixing matrix, say $\hat{B}$, such that $\hat{B}X$ is a good approximation to the original sources *S*. Let *B* = *A*^−1^ be the estimand of interest. Notationally following Hyvärinen et al. (2001), if *f*_*q*_ is the density for *S*(*q, v*) for *v* = 1, …, *V*, and *f* = (*f*_1_, …, *f*_*Q*_), then standard multivariate random variable transformation results imply that the joint density of *X*(·, *v*) is

$$\begin{array}{l} g\{X(\cdot ,\;v)\}=|\text{det}(B)|\prod_{q=1}^{Q}f_{q}\{S(q,v)\}\\ \;=|\text{det}(B)|\prod_{q\;=\;1}^{Q}f_{q}\{B(q,\;\cdot )X(\cdot ,\;v)\}, \end{array}$$

therefore the joint log-likelihood including all contributions for *v* = 1, …, *V* is

$$\begin{array}{l} ℒ\left(B,f\right)=\overset{V}{\underset{v=1}{\sum }}\overset{Q}{\underset{q=1}{\sum }}log\left[f_{q}\left\{B\left(q,\cdot \right)X\left(\cdot ,v\right)\right\}\right]+Vlog|det\left(B\right)|. \end{array}$$

It is generally not possible to solve the joint likelihood for the parameters in *f*_*q*_ and *B* simultaneously. Instead, an iterative optimization is often performed. Specifically, given the current estimate of *B* at iteration *k*, say ${\hat{B}}^{\left(k\right)}$, one can get an estimate for *S* via ${Ŝ}^{\left(k\right)}={\hat{B}}^{\left(k\right)}X$. Given *Ŝ*^(*k*)^(*q*, ·), density estimation techniques can be used to obtain ${\hat{f}}_{q}^{\left(k\right)}$, the estimate of ${\hat{f}}_{q}$ at iteration *k*.

We suggest the use of histogram smoothing as the density estimation technique, where the bin counts of the frequency histogram are assumed to follow a Poisson distribution and a penalized likelihood is constructed to produce a smooth density estimate. The details of histogram smoothing can be found in Eilers and Marx (1996), and we provide a sketch below. (Readers not familiar with statistical smoothing may skip the rest of this paragraph). Notationally, let *c*^(*k*)^(*q*, 0) < *c*^(*k*)^(*q*, 1) < … < *c*^(*k*)^(*q, J*) be equidistant histogram cutpoints, where *c*^(*k*)^(*q*, 0) = −ϵ + min *Ŝ*^(*k*)^(*q*, ·) and *c*^(*k*)^(*q, J*) = ϵ + max *Ŝ*^(*k*)^(*q*, ·). The number ϵ is added to avoid numerical boundary effects. Let $n^{\left(k\right)}\left(q,j\right)=\overset{V}{\underset{v=1}{\sum }}I\left\{c^{\left(k\right)}\left(j-1\right)<{Ŝ}^{\left(k\right)}\left(q,\cdot \right)\leq c^{\left(k\right)}\left(j\right)\right\}$, for *j* = 1, …, *J*, be the count of values between cutpoints *j* − 1 and *j* for row *q* of *Ŝ*^(*k*)^. Define the midpoints of intervals [*c*^(*k*)^(*q, j* − 1), *c*^(*k*)^(*q, j*)] by *m*^(*k*)^(*q, j*) for *j* = 1, …, *J*. We obtain a density estimate via the log-linear model *n*^(*k*)^(*q, j*) ~ Poisson{λ^(*k*)^(*q, j*)}, where $\log\left\{\lambda^{\left(k\right)}\left(q,\cdot \right)\right\}=\overset{L}{\underset{l=1}{\sum }}D^{\left(k\right)}\left\{m^{\left(k\right)}\left(q,\cdot \right),l\right\}\beta^{\left(k\right)}\left(q,l\right)$. Here the log function is presumed to act component-wise on vectors, *D*^(*k*)^ is a B-spline basis design matrix, *L* is the number of knots for B-splines, and β^(*k*)^(*q*, ·) is a vector of coefficients. To avoid overfitting the B-spline model, and to avoid sensitivity to the degrees of freedom, we choose a large value for the degrees of freedom and put a squared penalty on the coefficients. Let μ^(*k*)^(*q, j*) denote the expectation of *n*^(*k*)^(*q, j*), then the penalized log likelihood takes the form (Eilers and Marx, 1996)

$$\begin{array}{l} ℒ=\sum_{j\;=\;1}^{J}n^{\left(k\right)}(q,j)\ln\mu^{\left(k\right)}(q,j)-\sum_{j\;=\;1}^{J}\mu^{\left(k\right)}(q,j)\\ \;-\delta \sum_{l\;=\;3}^{L}\frac{{\left\{\Delta^{2}\beta^{\left(k\right)}(q,l)\right\}}^{2}}{2}, \end{array}$$

where δ is a parameter controlling the smoothness of the fit, Δ denotes the difference operator, Δ^2^β(·, *l*) = β(·, *l*) − 2β(·, *l* − 1) + β(·, *l* − 2). The resulting model is then a generalized linear mixed model on the counts. The B-spline basis is evaluated at the midpoint of the cutpoint interval. However, via interpolation, the smoother gives an estimate for all values, thus yielding a continuous function, say ${\hat{f}}_{q}^{\left(k\right)}\left(s\right)$, which is the density estimate.

Using generalized linear mixed models to penalize smoothing has become standard practice and is well described in Ruppert et al. (2003). Histogram smoothing as a density estimate appears to be less commonly used. However, we note that this p-spline based density smoother has very attractive properties (Eilers and Marx, 1996). First, it results in a proper density. Secondly, it elegantly handles boundary issues, unlike other density estimators (such as kernel density estimator). Thirdly, the estimated density conserves the first few empirical moments (means and variances) of the histogram, depending on the order of the B-splines. More details regarding these properties can be found in Eilers and Marx (1996). Note that, conservation of moments is an important property that guarantees the identifiability of the ICA model. We choose a cubic B-spline which then conserves the first two moments of the histogram.

Furthermore, due to the convenient differentiation properties of B-spline bases and the simple exponential (Poisson) model, the first and second derivatives of ${\hat{f}}_{q}^{\left(k\right)}$ are immediately available, where $d{\hat{f}}_{q}^{\left(k\right)}=\exp\left\{{\hat{f}}_{q}^{\left(k\right)}\right\}\beta^{\left(k\right)}\left(q,\cdot \right)dD^{\left(k\right)}$. Thus, derivatives of ℒ(*B*) are available in closed form, making gradient- and Hessian-based optimization algorithms easy to implement. This is useful for the stage of the algorithm for obtaining the next iterate of *B*. Accordingly, we use a Newton-Raphson method to update the mixing matrix. Specifically, let ℒ′ and ℒ″ denote the first and second derivatives of the log likelihood. At the *k*th iteration, we update *B* by

(2) $$\begin{array}{l} B^{\left(k+1\right)}=B^{\left(k\right)}-{ℒ}^{\prime \prime }{\left(B^{\left(k\right)}\right)}^{-1}{ℒ}^{\prime }\left(B^{\left(k\right)}\right). \end{array}$$

The starting values of *B* should satisfy the condition that the underlying ICs are the same for all subjects. Following Eloyan et al. (2013), we decompose the full matrix *X* using the population value decomposition *X* = *UΣV*^*T*^ (Crainiceanu et al., 2011), and the starting values of the *B*_*i*_ are chosen as the *i*th block of the rows of *UΣ*. Thus, given a starting value for *B*, histogram smoothing is used to obtain *f*_*q*_, then given the update for *f*_*q*_, the natural gradient algorithm is used to obtain *B* and these steps are iterated until convergence. Let *P* denote *B*^(*k*)^(*B*^(*k*+1)^)^−1^. We use the Amari metric between *B*^(*k*+1)^ and *B*^(*k*)^ as our convergence criterion (Amari, 1998), where the metric is defined as

$$\begin{array}{l} d\{B^{\left(k\right)},B^{\left(k\;+\;1\right)}\}=\frac{1}{2Q}\sum_{i\;=\;1}^{Q}\left(\sum_{j\;=\;1}^{Q}\frac{\left|P_{ij}\right|}{\max_{j}|P_{ij}|}-1\right)\\ \;+\frac{1}{2Q}\sum_{j\;=\;1}^{Q}\left(\sum_{i\;=\;1}^{Q}\frac{\left|P_{ij}\right|}{\max_{i}|P_{ij}|}-1\right). \end{array}$$

The Amari metric is useful, as it is invariant to permutation of the ordering of the ICs, a necessary condition for a convergence metric to be useful.

### 2.3. ICA based on parcellation

Most ICA algorithms (such as the commonly used fastICA, JADE, etc.) assume that ${\left\{S\left(q,v\right)\right\}}_{v=1}^{V}$ is an *i.i.d* collection for all *q* = 1, …, *Q*. Intrinsically, this is to assume that the values of the ICs are independent draws from a density. The *i.i.d* assumption is made for simplicity, but it may not hold for fMRI data. Calhoun et al. (2001b) considered possible violations of the independence assumption for task-based fMRI data. They found that the ICA algorithm was successful when the correlation in the signal was small, but it may fail when the signals are highly dependent. However, for most task-based fMRI and resting-state fMRI data, the correlation between voxels is negligible. Therefore, we do not pursue the approach to deal with violation of the independence assumption here. Instead, we consider relaxation of the identically distributed assumption.

Specifically, we propose to account for the difference in the activity across the brain by allowing different density distribution in different regions. To this end, we adopt the functional parcellation of the brain activity map proposed by Yeo et al. (2011). The parcellation includes 17 functional networks in the cerebral cortex, that is, *I* = 18 ROIs for the whole brain. We assume the signals are *i.i.d* within region but could be differently distributed across region. Under this assumption, the density function *f*_*q*_ can be written as the sum of the region-specific density function, that is,

$$\begin{array}{l} f_{q}\left(s\right)=\overset{I}{\underset{i=1}{\sum }}I\left(s\in R_{i}\right)f_{iq}\left(s\right), \end{array}$$

where *R*_*i*_ denotes the *i*th ROI, *f*_*iq*_ is the density function on *R*_*i*_. Thus, *f*_*iq*_ takes positive values on the *i*th region and zero elsewhere. The density estimate of *f*_*iq*_ can be obtained using the same procedure as proposed in Section 2.2, confined to the *i*th region. The estimate for *f*_*q*_ can be constructed by taking the sum of ${\hat{f}}_{iq}$. The rest of the ICA algorithm follows the proposed procedure in Section 2.2.

The proposed ICA algorithm can be summarized as follows:

_______________________________________________________________________________________________________

1. Choose an initial value of for the mixing matrix *B*.
2. Alternate until convergence of *B* using the Amari metric.
  - Let *S* = *BX*.
  - For each IC *q*, calculate the density function *f*_*iq*_(*s*) on the *i*th ROI, *i* = 1, 2, …, *I*, using the p-spline based density estimation algorithm.
  - Get $f_{q}\left(s\right)=\overset{I}{\underset{i=1}{\sum }}I\left(s\in R_{i}\right)f_{iq}\left(s\right)$.
  - Update the mixing matrix *B* using the Newton-Raphson method, see Equation (2).

_______________________________________________________________________________________________________

Note that, in the special case that *f*_1*q*_ = *f*_2*q*_ = … = *f*_*Iq*_, the above algorithm reduces to the algorithm proposed in Section 2.2 assuming *i.i.d* signals across the entire brain.

## 3. Simulation

We conduct simulation studies to evaluate the performance of the proposed ICA algorithm. We consider four settings where data are generated using different distributions. We compare the results of the proposed algorithm with fastICA (Hyvärinen et al., 2001), JADE (Cardoso, 1999), Pearson ICA (Karvanen et al., 2000), ProDenICA (Hastie and Tibshirani, 2002), and HDICA (Eloyan et al., 2013). We implement the algorithms fastICA, ProDenICA, JADE, PearsonICA using the R packages “fastICA” (Marchini et al., 2013), “ProDenICA” (Hastie and Tibshirani, 2010), “JADE” (Nordhausen et al., 2014), and “PearsonICA” (Karvanen, 2006). The proposed p-spline based ICA algorithm and the HDICA (Eloyan et al., 2013) are also implemented in R.

The computation environment is a multi-core Linux cluster with more than 680 cores running in the average of 2.5 GHz speed and 4.4 TB of memory. On average, the contrast-function based algorithms (fastICA, PearsonICA, JADE) perform much faster than the likelihood-based algorithms (p-spline ICA, ProDenICA, HDICA). (See Table 1 for a summary of the computation time using different algorithms.) However, since those are essentially two different sets of algorithms, we restrict the comparison of the computational intensity within the category of likelihood-based algorithm.

**Table 1:** **The average computation time (in seconds) per simulation replication using different algorithms in the simulation study**.

**Simulation**	**p-spline ICA**	**fastICA**	**Pearson ICA**	**JADE**	**ProDenICA**	**HDICA**
Scenario 1	6.21	< 0.01	0.01	0.01	3.12	308.34
Scenario 2	5.89	< 0.01	0.01	< 0.01	1.67	76.59
Scenario 3	7.27	0.01	0.01	0.01	1.74	123.84

In the first set of simulation studies, we assume there are *Q* = 3 independent components, and they are generated by *S*(1, ·) ~ Weibull(1, 1), *S*(2, ·) ~ Gamma(1, 1), and *S*(3, ·) ~ Gamma(2, 2), respectively. Standard Gaussian noises are added to the generated ICs. The mixing matrix is given by

$$A=\left(\begin{array}{ccc} 2 & 1 & 2\\ 3 & 3 & 1\\ 1 & 2 & 2 \end{array}\right).$$

**Figure 1:**
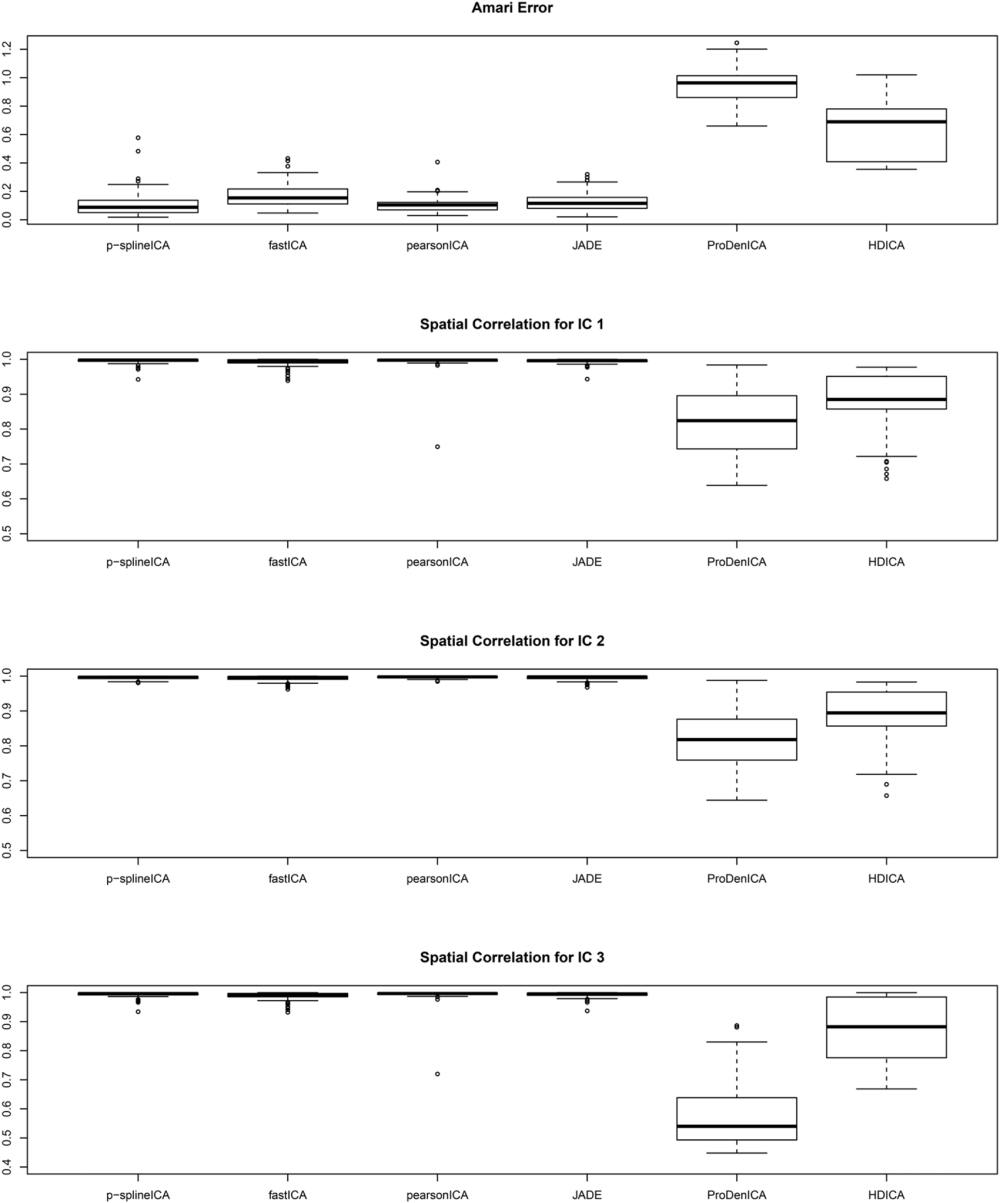
**Boxplots of the Amari errors and the spatial correlation calculated using different algorithms under simulation setting one**.

Figure 1 summarizes the simulation results based on 200 replications. The spatial correlation is the absolute correlation between the estimated spatial map and the true spatial map without noise. The Amari error is computed to evaluate the accuracy of the estimated mixing matrix (Amari, 1998). It is seen from the boxplots of the spatial correlation and the Amari errors that the proposed ICA algorithm performs equally well as fastICA, JADE, and PearsonICA, and all these algorithms perform substantially better than ProDenICA algorithm. ProDenICA fails probably due to the extreme values introduced by the noise (See more discussion in Risk et al., 2014). This shows that the ProDenICA is sensitive to extreme values, while our algorithm is robust to extreme values. The average computation time per replication is 6.21 s using the p-spline ICA, 3.12 s using ProDenICA, and 308.34 s using HDICA.

In the second setting, we assume the number of source signals *Q* = 2, and we generate the signals based on parcellation. Specifically, we partition the real line into 10 intervals, with cutoffs at the 10th, 20th, 30th, 40th, 50th, 60th, 70th, 80th, and 90th percentiles of the normal distribution. For the first IC, the density function is uniformly distributed within each interval, but the overall shape is approximately normal. For the second IC, the density function follows Laplace distribution within each interval, and the overall shape is approximately normal. The mixing matrix is given by

$$A=\left(\begin{array}{cc} 2 & 1\\ 3 & 2 \end{array}\right).$$

**Figure 2:**
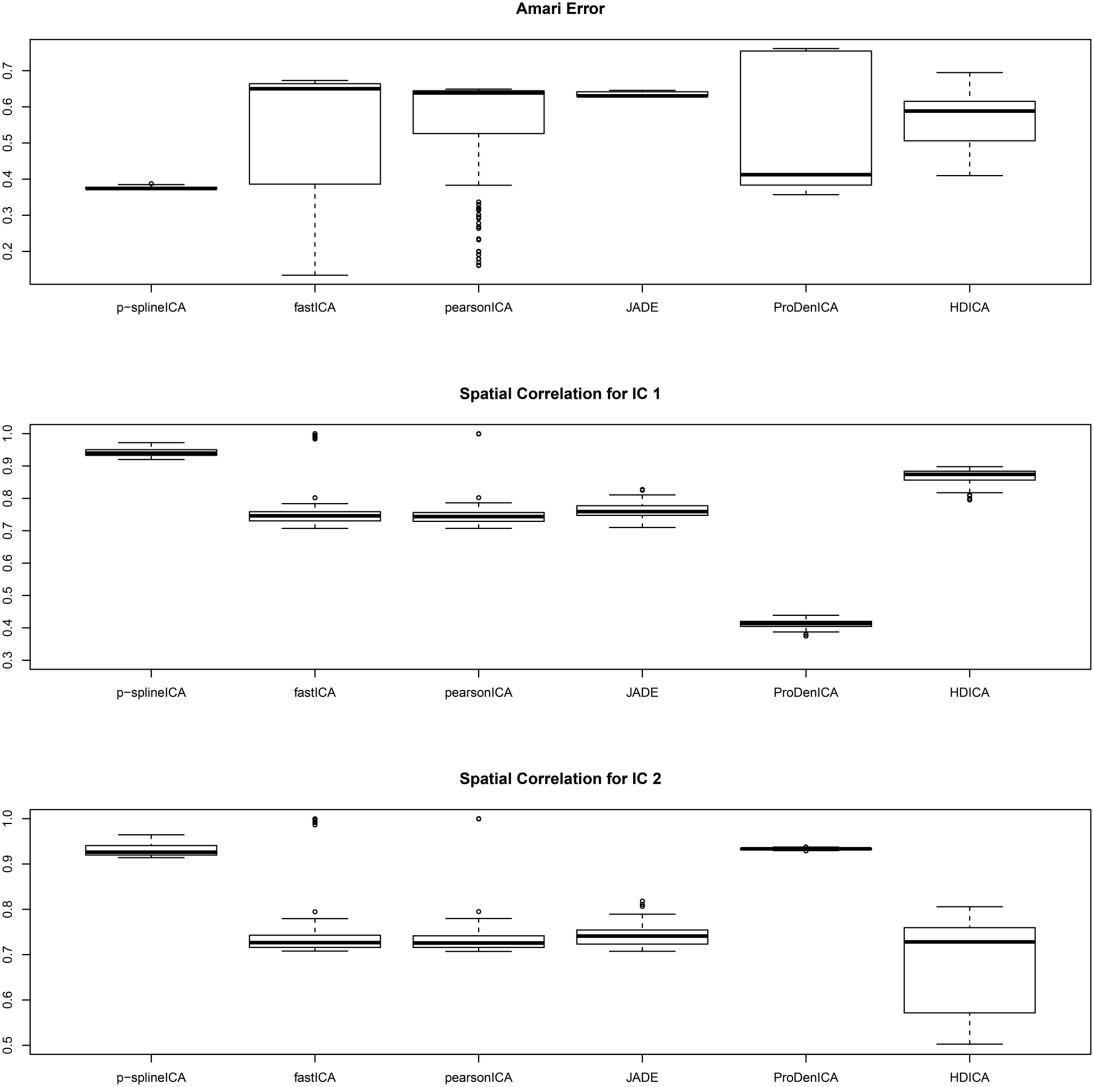
**Boxplotsof the Amari errors and the spatial correlation calculated using different algorithms under simulation setting two**.

The boxplots of the spatial correlation and the Amari errors based on 200 replications are summarized in Figure 2. Under the second scenario, the underlying signals have region specific densities, and the overall density functions for both components are approximately normal. All the competing algorithms considered in the simulation studies show substantial bias. These algorithms fail to recover the true signals because they heavily depend on the non-gaussianity assumption. On the contrary, the proposed algorithm accounts for the effect of parcellation and recovers the true signals with relatively high accuracy. The proposed algorithm substantially outperforms all the competing algorithms under the second setting. The average computation time per replication is 5.89 s using p-spline ICA, 1.67 s using ProDenICA, and 76.59 s using HDICA.

In the third setting, we generate multi-subject data with number of subject *I* = 3. The source signals are the same as those in the second setting, and the mixing matrices for the three subjects are given by

$$A_{1}=\left(\begin{array}{cc} 1 & 0.25\\ 0.5 & -0.5 \end{array}\right),\;\;A_{2}=\left(\begin{array}{cc} 1 & 2\\ 0.5 & -0.5 \end{array}\right),\;\;A_{3}=\left(\begin{array}{cc} 1 & 0.5\\ 0.5 & 2 \end{array}\right).$$

**Figure 3:**
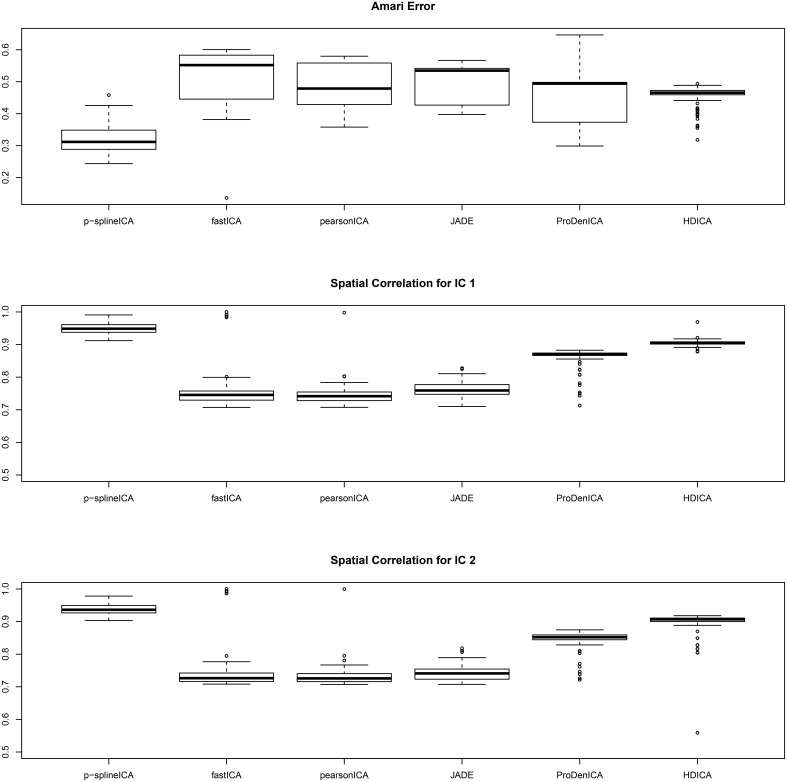
**Boxplots of the Amari errors and the spatial correlation calculated using different algorithms under simulation setting three**.

The simulation results are summarized in Figure 3, where in each simulation replication, the Amari error is calculated as the average of the Amari errors for all three subjects. The results show that, for multi-subject data, the proposed algorithm successfully recovers both the common spatial signals and the individual mixing matrices. In addition, for similar reasons as in the second setting, the proposed algorithm substantially outperforms all the competing algorithms.

**Figure 4:**
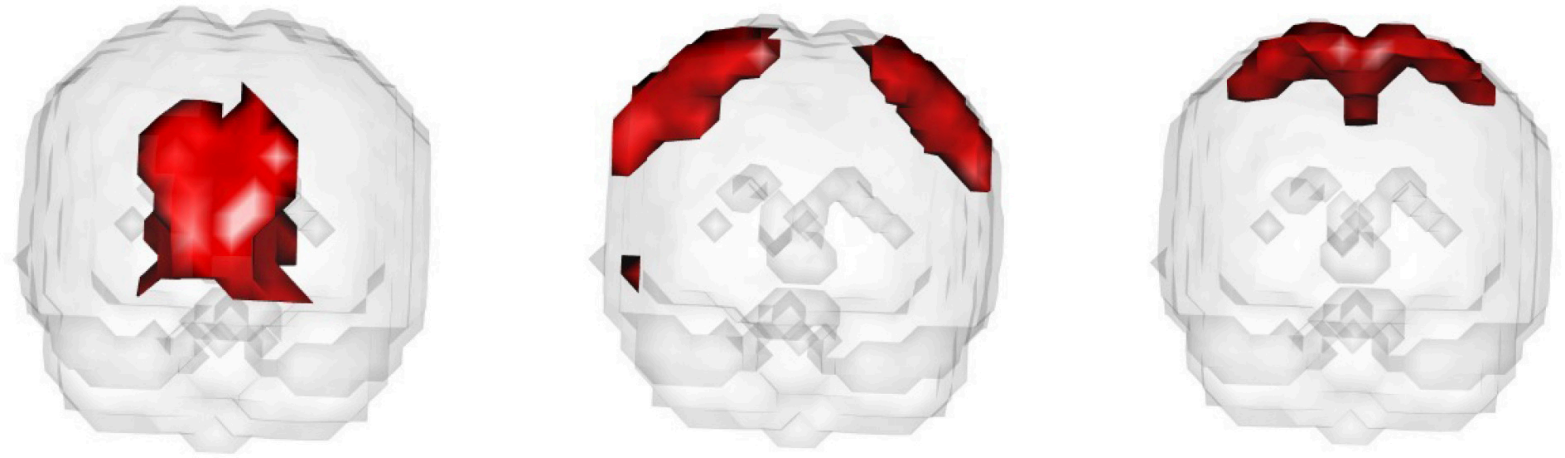
**The underlying signals for the fourth simulation setting: ICs 1, 2, and 3 (left to right)**.

**Figure 5:**
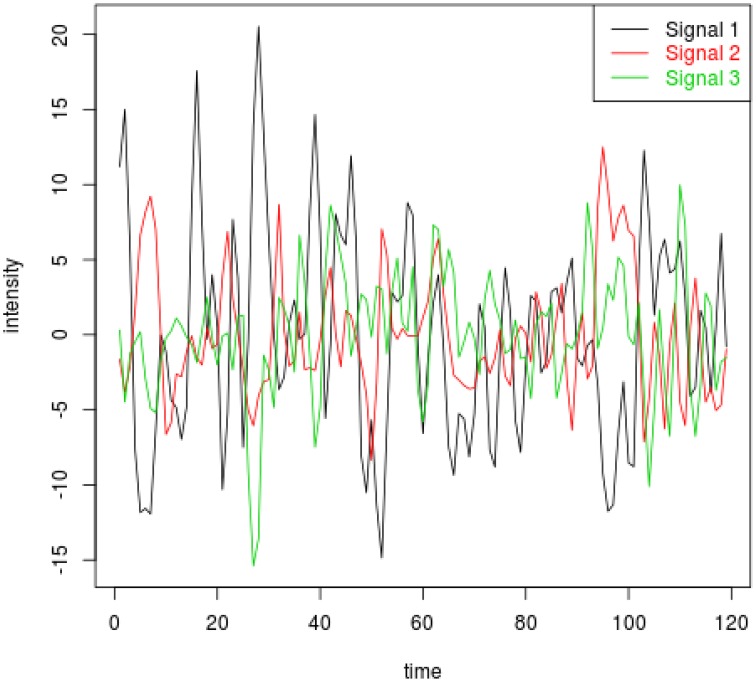
**Time courses corresponding to ICs 1, 2, and 3 for the fourth simulation setting**.

In the fourth setting, we generate the ICs and mixing matrices by mimicking signals from real fMRI data. Specifically, we run fastICA on 10 subjects from the NITRC 1000 Connectome dataset to get twenty ICs (networks). Three of the twenty networks are chosen as the true signals, and they are shown in Figure 4. The time courses are also signals from real data, obtained in a similar way as in Calhoun et al. (2009). They are shown in Figure 5. We first apply a two-stage dimension reduction using the method as described in Section 2.1. Then we apply the proposed atlas-based ICA algorithm using the brain parcellation proposed by Yeo et al. (2011). The correlation matrix between the true signals and the estimated signals using the proposed algorithm is

$$\left(\begin{array}{ccc} -0.999 & -0.025 & 0.007\\ 0.028 & 0.999 & -0.006\\ -0.004 & -0.003 & 0.999 \end{array}\right).$$

The results indicate that our proposed p-spline based ICA algorithm is successful in recovering signals from real fMRI data.

## 4. Application

We apply our proposed algorithm to the 1000 Functional Connectomes Project dataset, which consists of thousands of resting state scans combined across multiple sites with the goal of facilitating discovery and analysis of brain networks (Biswal et al., 2010). It is one of the largest freely available fMRI datasets. The fMRI scans were collected when the subjects stayed in the scanner for 2.2–20 min in resting state. Scanning parameters used to acquire the data from each site are detailed elsewhere (for complete information see https://www.nitrc.org/projects/fcon_1000/).

As the quality and scanning parameters vary across sites, we focus on data from the largest site, Cambridge, which contains *I* = 50 subjects. For the subjects used in this analysis, the number of time points is *T* = 119. We use the MNI template to remove the background noise and to retain voxels that are in the actual brain. For each subject, we have a *T* × *V* dimensional matrix *X*_*i*_. The group data matrix *X* is obtained by concatenating *I* subjects' data in the temporal domain.

**Figure 6:**
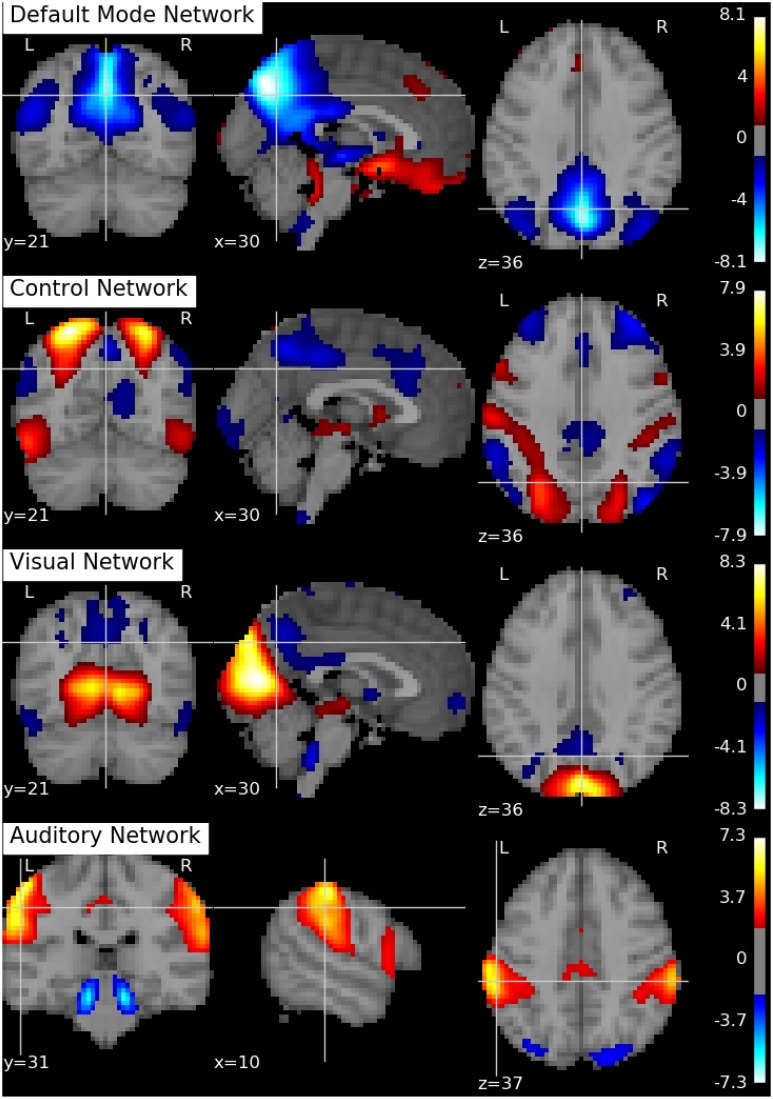
**Brain networks obtained by the p-spline ICA algorithm using data from the 1000 Functional Connectome Project**.

**Figure 7:**
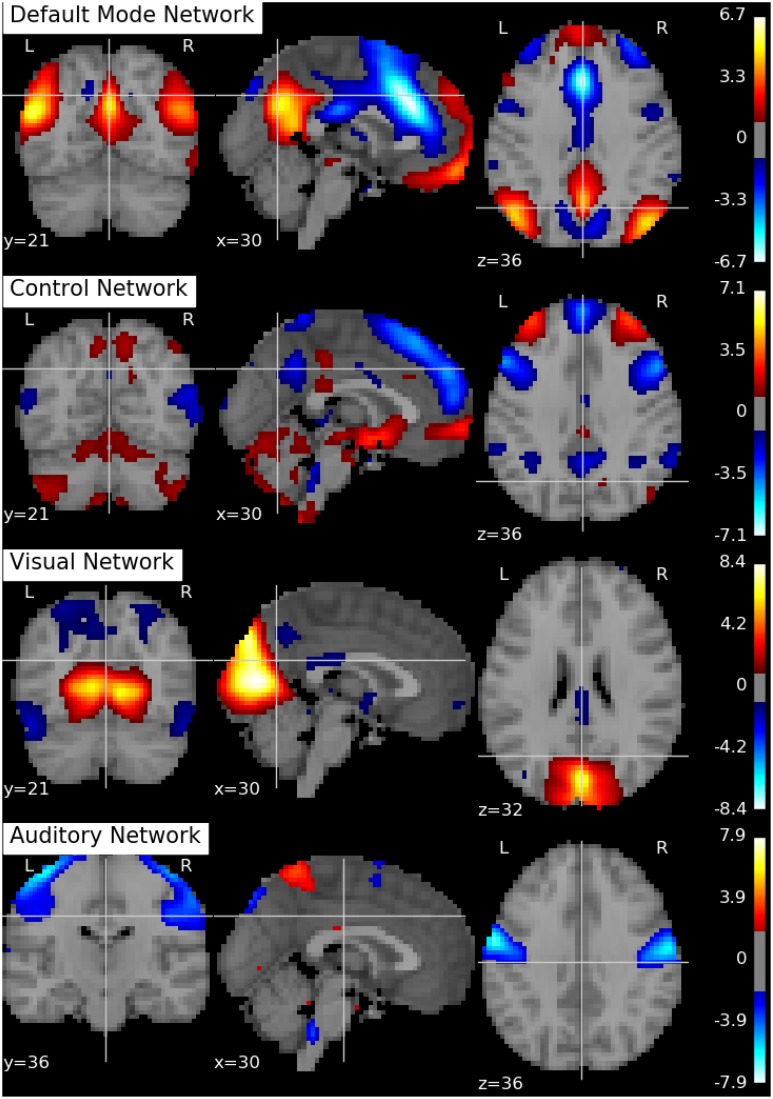
**Brain networks obtained by the fastICA algorithm using data from the 1000 Functional Connectome Project**.

Following Biswal et al. (2010), we assume there are *Q* = 20 independent components in this application. An SVD is performed to reduce the dimension of the aggregated data matrix to *Q* × *V*. The ICA algorithms are then applied to the reduced data matrix and the Python toolbox Nilearn (Abraham et al., 2014) is used for visualization of the estimation results. Specifically, the estimated ICs using the proposed p-spline based ICA algorithm are shown in Figure 6. Several main brain networks including the default mode network (DMN) and the control network are successfully identified by the proposed algorithm. As a comparison, the results from fastICA are shown in Figure 7. The ICs estimated by fastICA and the p-spline ICA are matched by correlation. Of the 20 pairs, the highest correlation is 0.99, the lowest correlation is 0.52, and the median correlation is 0.93. Specifically, the following is a list of these correlations for the major brain networks: visual network (0.99), auditory network (0.98), DMN (0.96), and control network (0.92).

As suggested by an anonymous reviewer, we investigate the impact of the dimension of the reduced space on the final results. Specifically, we select different values of *R* and *Q* (the number of eigenvectors in the subject-level and group-level dimension reduction), and rerun the ICA algorithm on the dimension reduced dataset. We set *R* = 15, 20, 30 and *Q* = 15, 20, 30, respectively. Similarly as in Li et al. (2007), we find that the IC estimates are well separated when *Q* = 15, 20. When *Q* = 30, the estimation of the major networks shows degradation and a few of the other estimated components seem to be noise. Specifically, the correlations for the major brain networks using *R* = 20, *Q* = 15, and *R* = 20, *Q* = 20 are as follows: visual network (0.96), auditory network (0.73), DMN (0.86) and control network (0.84). In addition, the correlations for the major brain networks using *R* = 20, *Q* = 30, and *R* = 20, *Q* = 20 are as follows: visual network (0.78), auditory network (0.61), DMN (0.88) and control network (0.69). In summary, we find that, although the estimation results depend on the number of components, the major networks appear to be robust against the choices of number of components.

## 5. Discussion

Independent component analysis is a factor-analytic approach that is commonly used in analyzing fMRI data. In this manuscript, we present a novel and simple ICA algorithm that is fast, likelihood based and straightforward to program. The algorithm is nonparametric, data-driven, and is blind to the particular distribution of the underlying signals. As a byproduct of the algorithm, we obtain the likelihood function of the ICA model which can be used for further statistical inference. It should be noted that, the likelihood function in our algorithm is a profile likelihood, since we are mainly interested in the mixing matrix estimates and the parameters over the spline basis are nuisance parameters. Indeed, one could also study the coefficients on the spline basis in a full likelihood, but this is not the goal of this manuscript, hence the variance of the estimator of the mixing matrix depends on the variance of the nuisance parameters.

The proposed algorithm is extended to allow for region specific IC density functions, on the rationale that most signals of interest are reasonably confined to a subset of the entire anatomical brain space (Guo and Pagnoni, 2008). When the source signals distribute identically across brain, the estimation accuracy of the parcellation-based estimator is similar to that of the full-brain estimator, because it becomes equivalent to the full-brain estimator. However, when the source signals distribute differently across brain, the full-brain estimator may result in substantial bias while the parcellation-based estimator can successfully recover the source signals. It should be noted that the parcellation based adjustment can be applied to other ICA algorithms as well. Indeed, for any gradient-based ICA, one can do the adjustment by taking a weighted sum over the updates of each of the parcellations, where the weights account for the number of samples in the parcellations. This flexibility ensures the generalizability of the proposed parcellation based adjustment.

Simulation studies show that our proposed algorithm works well in both the simple and complex situations, and it substantially outperforms the existing ICA algorithms when the identically distributed assumption of the source signals is violated. In applying the proposed algorithm to the fMRI data, we choose to account for the difference in brain activities across regions by using the brain parcellation proposed by Yeo et al. (2011). Our data application results show that the proposed algorithm successfully identifies the main brain networks in the 1000 Functional Connectomes Project dataset.

There are a few directions for future research. Firstly, the test-retest reliability of the intrinsic brain networks is an important issue and has been studied extensively in recent years. For example, Zuo et al. (2010) found that a few functionally relevant components (such as the default mode, auditory-motor and executive control) show the highest reliability across all components. It would be interesting to compare different ICA algorithms in identifying and characterizing those functionally relevant components. Secondly, there are a variety of existing brain parcellation schemes, including those proposed by Tzourio-Mazoyer et al. (2002); Fischl et al. (2004); Beckmann et al. (2009); Yeo et al. (2011). It would be interesting to study the optimal choice of parcellation under different scientific scenarios. Thirdly, as pointed out by an anonymous reviewer, pre-whitening, although a standard pre-processing procedure, may result in loss of information and bias in estimation (Cardoso, 1994). It would be interesting to investigate alternative pre-processing procedures to avoid the bias introduced by pre-whitening.

### Conflict of interest statement

The authors declare that the research was conducted in the absence of any commercial or financial relationships that could be construed as a potential conflict of interest.
